# Ribosomal Internal Transcribed Spacer of *Prototheca wickerhamii* Has Characteristic Structure Useful for Identification and Genotyping 

**DOI:** 10.1371/journal.pone.0081223

**Published:** 2013-11-27

**Authors:** Noriyuki Hirose, Kazuko Nishimura, Maki Inoue-Sakamoto, Michiaki Masuda

**Affiliations:** 1 Department of Microbiology, Dokkyo Medical University School of Medicine, Tochigi, Japan; 2 Fukushima Plant, BD Japan, Co., Ltd., Fukushima, Japan; 3 First Laboratories, Co. Ltd., Kanagawa, Japan; 4 Dermatology Division, Amakusa Chuo General Hospital, Kumamoto, Japan; 5 Department of Dermatology and Plastic Surgery, Faculty of Life Sciences, Kumamoto University, Kumamoto, Japan; University of Mississippi Medical Center, United States of America

## Abstract

*Prototheca* species are achlorophyllous algae ubiquitous in nature and known to cause localized and systemic infection both in humans and animals. Although identification of the *Prototheca* species in clinical specimens is a challenge, there are an increasing number of cases in which molecular techniques have successfully been used for diagnosis of protothecosis. In this study, we characterized nuclear ribosomal DNA (rDNA) of a strain of *Prototheca* (FL11-0001) isolated from a dermatitis patient in Japan for its species identification. When nuclear rDNA of FL11-0001 and that of various other *Prototheca* strains were compared by polymerase chain reaction (PCR), the results indicated that the sizes of ribosomal internal transcribed spacer (ITS) were different in a species-dependent manner, suggesting that the variation might be useful for differentiation of *Prototheca* spp. Especially, ITS of *P. wickerhamii*, the most common cause of human protothecosis, was distinctively larger than that of other *Prototheca* spp. FL11-0001, whose ITS was comparably large, could easily be identified as *P. wickerhamii*. The usefulness of the PCR analysis of ITS was also demonstrated by the discovery that one of the clinical isolates that had previously been designated as *P. wickerhamii* was likely a novel species. Furthermore, our data demonstrated that nucleotide sequences of *P. wickerhamii* ITS are heterogenous between different rDNA copies in each strain and also polymorphic between strains. Phylogenetic analysis suggested that the ITS sequences could be classified to four clades, based on which *P. wickerhamii* strains might be grouped into at least two genotypes. Comprehensive characterization of *Prototheca* rDNA may provide valuable insights into diagnosis and epidemiology of protothecosis, as well as evolution and taxonomy of *Prototheca* and related organisms.

## Introduction

*Prototheca* species are achlorophyllous algae closely related to *Chlorella* and ubiquitous in nature [1-3]. Currently, it is generally accepted that six species belong to the genus *Prototheca*; namely, *P. wickerhamii*, *P. zopfii*, *P. blaschkeae*, *P. cutis*, *P. ulmea* and *P. stagnora* [3-5] of which the first four species have been shown to cause infection in animals, such as cattle and dogs [6-9], and humans [2,10,11]. Recently, an increasing number of human cases of *Prototheca* infection have been reported, including opportunistic infection in immune compromised individuals [2,11]. Therefore, diagnosis of protothecosis and identification of the causative *Prototheca* species are becoming more and more important.

Clinical diagnoses of protothecosis have traditionally been made by histopathological examination of affected tissues and morphological and biochemical examinations of the isolated organism [2,10]. However, these methods tend to generate equivocal results, and it has often been challenging to determine the causative *Prototheca* species [12,13]. Therefore, it is possible that there have been cases in which the identification was not definitive, although *P. wickerhamii* among the pathogenic *Prototheca* spp. appears to be the most common cause for human infection [2,11]. Recently, it has been shown that molecular techniques, such as polymerase chain reaction (PCR) genotyping using species- and genotype-specific primers, nucleotide sequencing of ribosomal DNA (rDNA), proteomic analysis, and real-time PCR could be useful for identification of *Prototheca* spp. [13-20].

**Figure 1:**
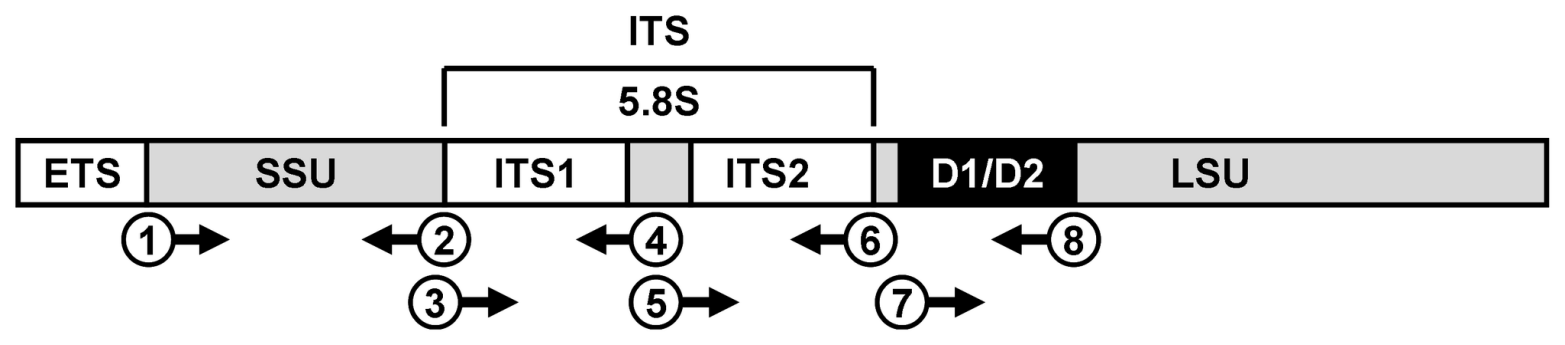
Schematic diagram of the transcriptional unit of rDNA. Positions of external transcribed spacer (ETS), small subunit rDNA (SSU), internal transcribed spacer (ITS) comprising ITS1, 5.8S rDNA and ITS2, and large subunit rDNA (LSU) with its D1/D2 domain are shown. Numbers in the circle correspond to the primers listed in Table 3. Upstream and downstream primers are indicated by arrows toward right and left, respectively.

Genomes of eukaryotic organisms generally contain multiple copies of nuclear rDNA transcriptional units, each of which consists of external transcribed spacer (ETS), 18S small subunit (SSU) rDNA, ITS and 26S/28S large subunit (LSU) rDNA (Figure 1). The ITS is further divided into ITS1, 5.8S rDNA and ITS2 (Figure 1). The LSU rDNA harbors the D1/D2 domain comprising two adjacent variable regions D1 and D2 (Figure 1). Nucleotide sequencing of the D1/D2 domain has previously been used for identification of *P. wickerhamii* [13-15]. In this study, in the course of identifying a clinically isolated strain of *Prototheca* (FL11-0001), we attempted to develop and evaluate additional molecular methods for differentiating and characterizing *Prototheca* spp. Our results indicate that PCR analysis of the ITS of nuclear rDNA could serve as a simple and reliable method for differentiating *P. wickerhamii* from other *Prototheca* spp. As a proof of principle using this method, we discovered that a clinical strain which had previously been designated as *P. wickerhamii* [21] was in fact not this species, but perhaps a novel member of genus *Prototheca*. We also demonstrate that nucleotide sequences of *P. wickerhamii* ITS are heterogeneous between different rDNA copies in each strain and also polymorphic between strains and that the polymorphism might be useful for intraspecies genotypic classification.

## Results

### Identification of strain FL11-0001 as *P. wickerhamii*

**Figure 2:**
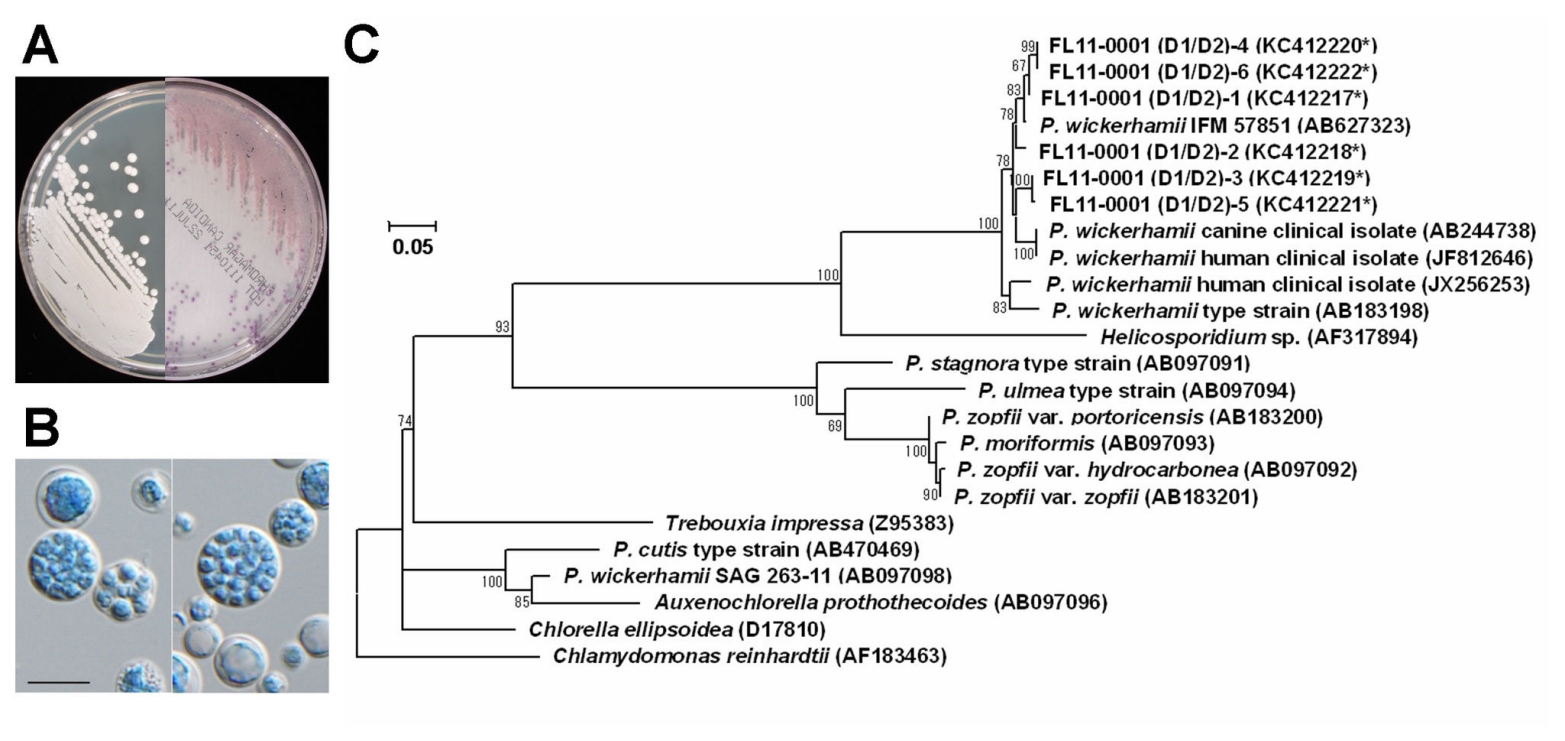
Characterization of the *Prototheca* clinical strain FL11-0001. (a) Colony appearance of FL11-0001. Left and right panels show colonies grown on PDA at 25°C for 7 days and CAC at 35°C for 4 days, respectively. (b) Cellular morphology of FL11-0001. Scale bar corresponds to 10 μm. (c) Phylogenetic tree of the nucleotide sequences of LSU D1/D2 region. The GenBank accession number of each sequence is shown in the parenthesis, and the accession numbers with an asterisk indicate the new sequences determined in this study from cloned DNA. The evolutionary history was inferred by using the Maximum Likelihood method with the Tamura 3-parameter model [27] selected based on the BIC scores. Additionally, a discrete Gamma distribution was used to model evolutionary rate differences among sites, and the rate variation model allowed for some sites to be evolutionarily invariable. The tree with the highest log likelihood is drawn to scale, and the scale bar (0.05) is shown in the unit of base substitutions per site. The bootstrap values less than 50 are not shown. The outgroup is *Chlamydomonas* *reinhardtii*.

FL11-0001 is a clinical isolate of *Prototheca* derived from a dermatitis patient in Japan. On both potato dextrose agar (PDA) and CHROMagar *Candida* (CAC), it produced yeast-like colonies (Figure 2A). Microscopically, FL11-0001 had spherical to subspherical sporangia (7.4 to 13.0 μm in diameter) containing two, four or a larger number of sporangiospores (2.6 to 4.3 μm) (Figure 2B). Biochemical tests with API 20C AUX indicated that FL11-0001 assimilated glucose, glycerol, galactose and trehalose, generating the code number of 6040040. These results were compatible with the possibility that FL11-0001 was either *P. wickerhamii* [1,2,10] or recently discovered *P. cutis* [5].

For definitive identification of FL11-0001, we determined the nucleotide sequence of its LSU D1/D2. When the PCR-amplified DNA was directly used as a template, the data contained a considerable amount of ambiguity, strongly suggesting that FL11-0001 has multiple copies of nuclear rDNA with heterogeneous D1/D2 sequences. In order to determine the D1/D2 sequences of the individual copies, the amplified DNA was molecularly cloned and sequenced. The results showed that randomly selected six clones, FL11-0001(D1/D2)-1 through -6, varied in their sequence with 94 to 100 % similarities to each other. Phylogenetic analysis showed that all of the determined D1/D2 sequences of FL11-0001 were clustered with those of other *P. wickerhamii* strains (Figure 2C), showing that FL11-0001 could definitively be identified as *P. wickerhamii*.

### PCR analysis of rDNA

**Figure 3:**
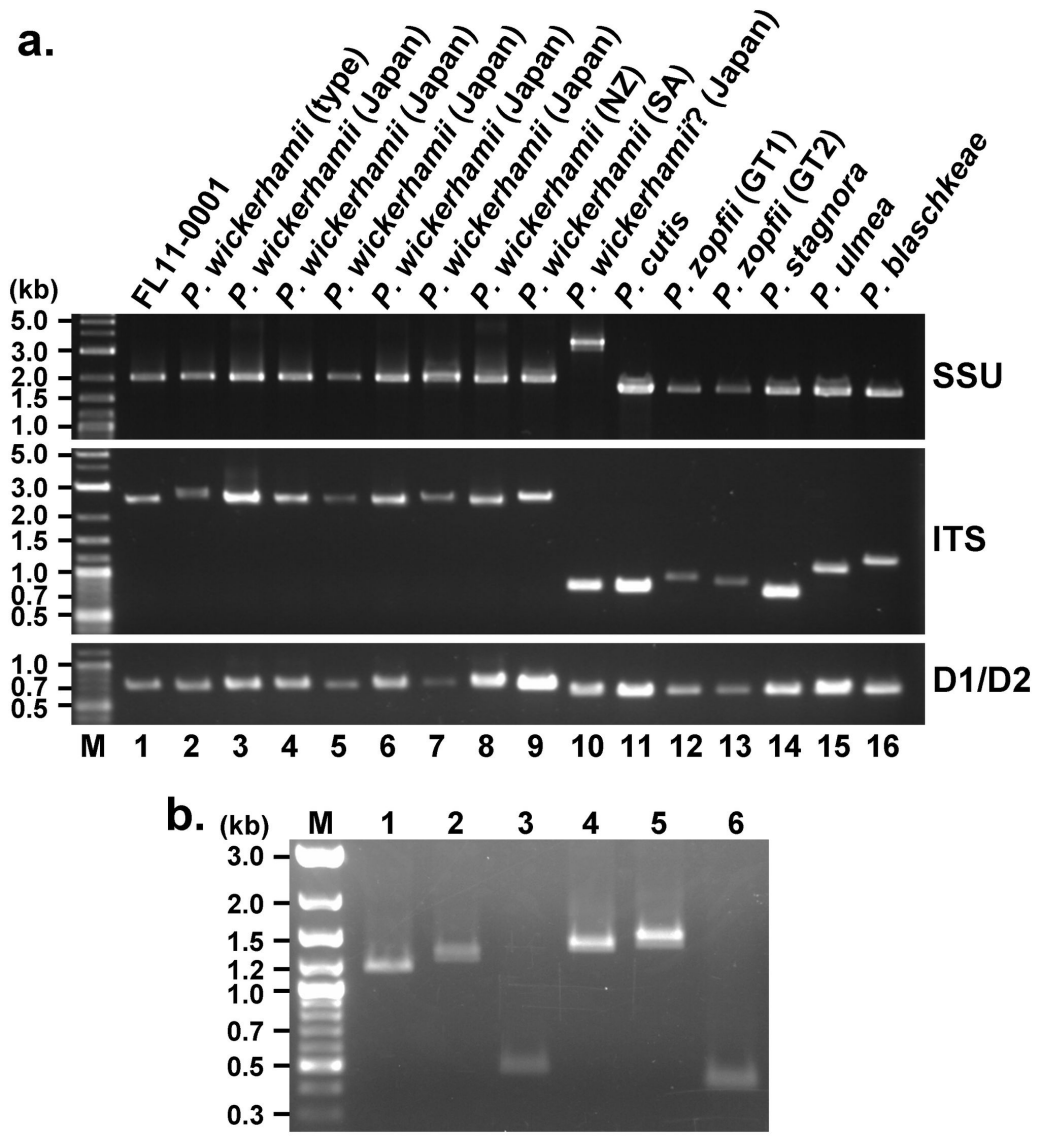
Electrophoretic patterns of the PCR products. (a) PCR products of the SSU rDNA, ITS which includes ITS1, 5.8S rDNA and ITS2, and LSU D1/D2. Lanes. M, size markers; 1, FL11-0001; 2, IFM 56739^T^; 3, IFM 57851; 4, IFM 58036; 5, IFM 52823; 6, IFM 58159; 7, DMP 12-01; 8, JCM 9643; 9, JCM 9644; 10, IFM 53848; 11, JCM 15793^T^; 12, SAG 2063^T^; 13, NUBS 5; 14, JCM 9641^T^; 15, ATCC 50112^T^; 16, SAG 2064^T^. Designations of the strains used are shown at the top for convenience. NZ, New Zealand; SA, South Africa; GT1, genotype 1; GT2, genotype 2. Question mark in lane 10 indicates that the initial designation is questionable. (b) PCR products of the ITS1 (lanes 1 through 3) and ITS2 (lanes 4 through 6). Lanes: M, size markers; 1 and 4, FL11-0001; 2 and 5, type strain of *P. wickerhamii* (IFM 56739^T^); 3 and 6, *P. zopfii* genotype 1 (SAG 2063^T^).

In the course of identifying FL11-0001, PCR products of its SSU rDNA, ITS and D1/D2 (Figure 1) were also compared with those of various other *Prototheca* strains (Figure 3A). The product sizes of SSU rDNA, ITS and D1/D2 of FL11-0001 (Figure 3A, lane 1) were comparable to those of the environment-derived type strain (Figure 3A, lane 2), as well as seven other clinical strains (Figure 3A, lanes 3 through 9), of *P. wickerhamii*. The sizes of ITS, which includes ITS1, 5.8S rDNA and ITS2 (Figure 1), appeared to vary in a species-dependent manner, and the ITS of *P. wickerhamii* (Figure 3A, lanes 1 through 9) was distinctively larger than that of other *Prototheca* spp. (Figure 3A, lanes 11 through 16). Intriguingly, the ITS of IFM 53848, which had previously been designated as *P. wickerhamii* [21], was too small for that of *P. wickerhamii* (Figure 3A, lane 10), while its SSU rDNA was clearly larger than that of *P. wickerhamii* or any other known *Prototheca* spp. We also performed PCR to compare sizes of ITS1 and ITS2 of *P. wickerhamii* with those of *P. zopfii* (Figure 3B). Sizes of both ITS1 and ITS2 of *P. wickerhamii* were distinctively larger than those of *P. zopfii*. Notably, there were subtle differences in the PCR data between FL11-0001 and the type strain IFM 56379^T^ (Figure 3B, lanes 1 and 2 for ITS1 and lanes 4 and 5 for ITS2).

### Sequence polymorphism of *P. wickerhamii* ITS

**Figure 4:**
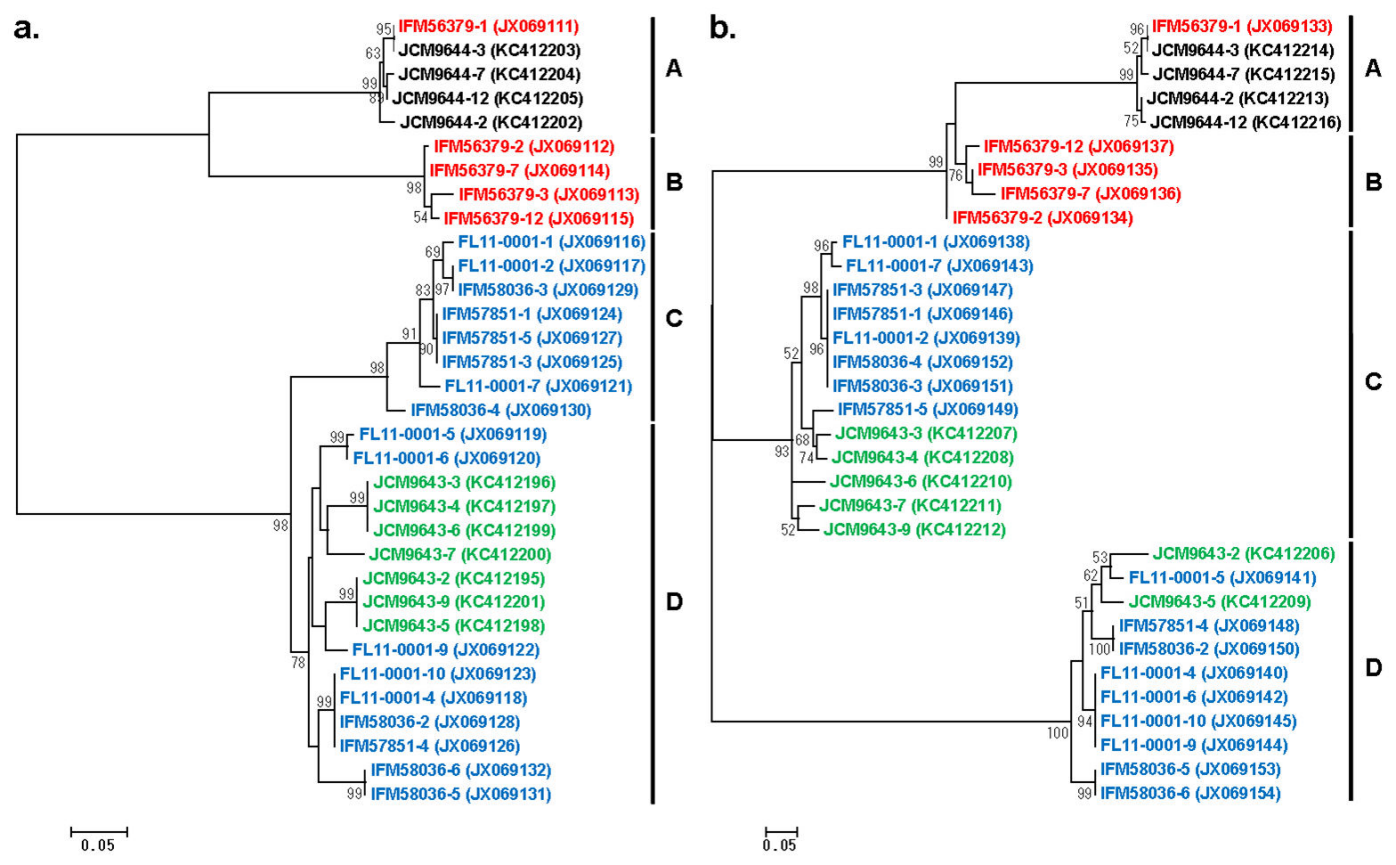
Unrooted phylogenetic trees of ITS1 and ITS2 sequences derived from various strains of *P. wickerhamii*. The 400-bp sequences corresponding to the 5’ end of ITS1 (a) and the 3’ end of ITS2 (b) were determined in this study from cloned DNA, and their accession numbers are indicated in the parentheses. The evolutionary history was inferred by using the Maximum Likelihood method with the Hasegawa-Kishino-Yano model [28] selected based on the BIC scores. A discrete Gamma distribution was also used to model evolutionary rate differences among sites. The trees with the highest log likelihood are drawn to scale, and the scale bars (0.05) are shown in the unit of base substitutions per site. The bootstrap values less than 50 are not shown. Four sequence clades expediently designated as A through D are indicated. Red sequences are derived from the type strain isolated in U. S. A. Black, blue and green sequences are derived from clinical strains isolated in South Africa, Japan, and New Zealand, respectively.

In order to characterize the uniquely large ITS of *P. wickerhamii*, we first attempted to determine the nucleotide sequence of FL11-0001 ITS by directly using the PCR product as a template. However, the results contained numerous nucleotide sequence ambiguities, suggesting that the ITS sequences might be heterogenous between different copies of rDNA in a single strain of *P. wickerhamii*. To compare the ITS sequences of individual copies of rDNA, the ITS PCR products of various *P. wickerhamii* strains (FL11-0001, IFM 56379^T^, IFM 57851, IFM 58036, JCM 9643 and JCM 9644) were cloned and the sequences of randomly selected clones were determined. The results revealed that the ITS sequences were heterogeneous between different rDNA copies within each strain of *P. wickerhamii* and also polymorphic between strains. Phylogenetic analyses by using 400-bp sequences in the 5’ and 3’ ends of ITS1 and ITS2, respectively, both suggested that the ITS sequences of *P. wickerhamii* were grouped into four clusters that were expediently designated as clades A through D (Figure 4). The ITS sequences of the type strain (IFM 56379^T^) belonged to clades A and B, while those of a clinical isolate JCM 9644 belonged to only clade A. On the other hand, the ITS sequences of three other clinical isolates (FL11-0001, IFM 57851 and IFM 58036) belonged to clades C and D. In most clones, their ITS1 and ITS2 sequences are in the same clade (Figure 4). However, there were clones, such as JCM9643-3, 4, 6, 7 and 9 derived from a clinical isolate JCM 9643, whose ITS1 sequences belonged to clade D (Figure 4A), whereas their ITS2 sequences were found in clade C (Figure 4B). Variation between the sequences of different strains in a single clade was smaller than that between the sequences of the same strain in different clades (Figure 4).

#### Structure of *P. wickerhamii* ITS

In order to characterize the structure of *P. wickerhamii* ITS more in detail, we determined the entire sequences of the ITS molecular clones, IFM 56379-1, -2, FL11-0001-1 and -6, representing clades A through D, respectively (Figure 4). Notably, the sizes of ITS1, 5.8S rDNA and ITS2 deduced from the nucleotide sequences (Table 1) are compatible with the subtle differences in the PCR data between FL11-0001 and the type strain IFM 56379^T^ (Figure 3B). BLASTn searches by using the obtained ITS1 or ITS2 sequences did not detect any other similar sequences in the GenBank database as of the date of submission of this paper.

**Table 1:** Sizes of ITS1, 5.8S rDNA and ITS2 of *P. wickerhamii* ITS clones.

Strain	Clone	Accession No.	Clade	Size (bp)		
				ITS1	5.8S rDNA	ITS2
IFM 56379^T^	IFM56379-1	KC412193	A	1,293	181	1,196
	IFM56379-2	KC412194	B	1,304	177	1,312
FL11-0001	FL11-0001-1	JX499274	C	1,079	176	1,170
	FL11-0001-6	JX069110	D	1,050	177	1,243

## Discussion

In this study, we compared various *Prototheca* spp. for their rDNA by PCR and nucleotide sequencing in the course of identifying a clinical strain of *Prototheca* isolated in Japan. Whereas LSU D1/D2 sequences have previously been used for identifying *P. wickerhamii* [13-15], we found that direct sequencing of the D1/D2 PCR products generated ambiguous results. In order to collect unequivocal data, we took a strategy of cloning the D1/D2 PCR products and determining the sequence of individual clones. The results strongly suggested that each individual strain of *P. wickerhamii* has multiple copies of nuclear rDNA with heterogeneous D1/D2 sequences, although exact number of the copies and their genomic organization are unknown. While the LSU D1/D2 sequences are generally useful for species identification because of their invariance in each species [22], similarities between the D1/D2 sequences of *P. wickerhamii* listed in public databases are relatively low (93 to 95% except for 100% identity between AB244738 and JF812646) for a single species. This may be due to the inter-copy heterogeneity revealed in this study. The heterogeneity of the D1/D2 sequneces found in this study is also comparable with the previous report that the SSU rDNA sequences of a single strain of *P. wickerhamii* are variable [23]. Heterogeneity between different copies of rDNA has previously been documented for other eukaryotic microorganisms, such as *Plasmodium* spp., with its functional significance [24]. However, it is unknown yet why and how the D1/D2 sequences of *P. wickerhamii* evolved the heterogeneity. 

Importantly, it was shown in this study that sizes of ITS of pathogenic *Prototheca* spp. were variable in the order of *P. wickerhamii* > *P. blaschkeae* > *P. zopfii* > *P. cutis*. Especially, the uniquely large ITS (about 2.6 kb) of *P. wickerhamii*, the most common cause of human protothecosis, may be useful for discriminating this species from other *Prototheca* spp. While the state-of-the-art molecular techniques, such as PCR genotyping using species- and genotype-specific primers, proteomic analysis, and real-time PCR, have successfully been applied for identification of *Prototheca* spp. [16-20], rDNA profiling based on conventional PCR data as used in this study may still serve as a simple and inexpensive method in regular clinical settings. Usefulness of the rDNA PCR was typically demonstrated by our discovery that a clinical isolate IFM 53848 previously designated as *P. wickerhamii* [21] was likely a different species. While the ITS of IFM 53848 was clearly smaller than that of *P. wickerhamii*, its SSU rDNA (>3.0 kb) was distinctively larger than that of any other species currently listed in the genus *Prototheca*. Therefore, it is possible that IFM 53848 represents a novel *Prototheca* species. To test this possibility, further studies are in progress to characterize this strain.

 To our knowledge, the present study is the first to describe the heterogeneity of ITS sequences between different copies of rDNA within each strain of *P. wickerhamii* and also the ITS polymorphism between different strains. It is unknown how the ITS sequences of *P. wickerhamii* became variable. However, it is unlikely that the variation was generated simply by random mutation of an ancestral ITS sequence, because our results indicated that the ITS sequences could be grouped into four distinct clades, A through D. While the type strain IFM 56379^T^ was shown to have the ITS sequences belonging to only clades A and B, all of the Japanese strains examined (FL11-0001, IFM 57851 and IFM 58036) had the ITS of only clades C and D (Figure 4). Therefore, it is possible that *P. wickerhamii* strains can be classified into at least two genotypes depending on which clades of ITS they have; namely, genotype 1 represented by the type strain IFM 56379^T^ with ITS sequences of clades A and B and genotype 2 represented by FL11-0001 with ITS sequences of clades C and D. The strain from South Africa (JCM 9644) whose ITS sequences belonged to only clade A may be classified to genotype 1, since analysis of additional clones may reveal the presence of clade B sequences in this strain. The phylogenetic trees suggest that the strain from New Zealand (JCM 9643) is genotype 2, although there were clones, such as JCM9643-3, 4, 6, 7 and 9 derived from this strain, whose ITS1 and ITS2 were classified to clades D and C, respectively. Thus, there seem to be at least three kinds of combination of ITS1 and ITS2 in genotype 2; namely, ITS1^C^-ITS2^C^, ITS1^D^-ITS2^D^, and ITS1^D^-ITS2^C^. The exact mechanism for generation of the apparent discordance between ITS1 and ITS2 is unknown. However, DNA recombination between different rDNA copies, among other possibilities, might be responsible. *P. zopfii* has been classified to genotypes 1 and 2 based on the difference in the SSU rDNA sequences [4]. However, SSU-based intraspecies genotyping of *P. wickerhamii* would be difficult because SSU rDNA of *P. wickerhamii* are very variable, resulting in at least 17 different groups in a single strain [23]. Our results suggest that sequence determination of a few ITS clones derived from a strain of *P. wickerhamii* combined with the phylogenetic analysis of the obtained data by using the ITS sequences determined in this study might be able to decide genotype of the strain. Although it is unlikely that the three Japanese patients, who FL11-0001, IFM 57851 and IFM 58036 were isolated from, had been infected from the same source, all of the three strains belong to genotype 2. One strain isolated in New Zealand (JCM 9643) also appears to belong to genotype 2. It leads to a speculation that *P. wickerhamii* of genotype 2 might be more common in the Asia-Pacific region than genotype 1, which might be the major one in America (e.g., IFM 56379^T^) and Africa (e.g., JCM 9644). Extensive analysis of the ITS sequences of the P. *wickerhamii* strains isolated from various geographical locations may elucidate the relationship between geographical distribution and genotypes of this species. The findings in this study in combination with further characterization of rDNA sequences of *Prototheca* spp. and related organisms may also provide valuable insights into molecular evolution and taxonomy of Trebouxiophyceae, Chlorophyta.

## Materials and Methods

### *Prototheca* strains

*Prototheca* strains used in this study are listed in Table 2. FL11-0001 was isolated from a patient with cutaneous protothecosis in Kumamoto, Japan. DMP12-01 and NUBS 5 [25] were kindly provided by Keiko Nishimura (Okayama Medical Centre, National Hospital Organization) and Dr. Rui Kano (Nihon University School of Veterinary Medicine), respectively. Other strains were obtained from the Medical Mycology Research Center at Chiba University, RIKEN BioResource Center, American Type Culture Collection and the Culture Collection of Algae at the University of Göttingen. Upon receipt, the cells were grown on PDA (Nissui, Japan) at 25°C.

**Table 2:** Strains used in this study.

Strain no.	Initial designation	Source
FL11-0001	Unknown	Cutaneous protothecosis (Kumamoto, Japan)
IFM 56379^T^	*P. wickerhamii* (type strain)	Lavatory drain (Illinois, U. S. A.)
IFM 57851	*P. wickerhamii*	Cutaneous protothecosis (Hiroshima, Japan)
IFM 58036	*P. wickerhamii*	Cutaneous protothecosis (Tokyo, Japan)
IFM 52823	*P. wickerhamii*	Cutaneous protothecosis (Okinawa, Japan)
IFM 58159	*P. wickerhamii*	Cutaneous protothecosis (Okinawa, Japan)
DMP12-01	*P. wickerhamii*	Blood (Okayama, Japan)
JCM 9643	*P. wickerhamii*	Blood, skin, peritoneum (New Zealand)
JCM 9644	*P. wickerhamii*	Breast and bones (South Africa)
IFM 53848	*P. wickerhamii*	Systemic protothecosis (Hiroshima, Japan)
JCM 15793^T^	*P. cutis* (type strain)	Cellulitis-like dermatitis (Chiba, Japan)
SAG 2063^T^	*P. zopfii* (GT 1) (type strain)	Cattle liquid manure (Germany)
NUBS 5	*P. zopfii* (GT 2)	Bovine mastitis (Japan)
JCM 9641^T^	*P. stagnora* (type strain)	Sludge (Ohio, U. S. A.)
ATCC 50112^T^	*P. ulmea* (type strain)	Slime flux of *Ulmus americana* (Pennsylvania, U. S. A.)
SAG 2064^T^	*P. blaschkeae* (type strain)	Onychomycosis (Germany)

IFM: Medical Mycology Research Centre, Chiba University. JCM: RIKEN BioResource Centre. ATCC: American Type Culture Collection. SAG: The Culture Collection of Algae at the University of Göttingen. GT: Genotype

### Morphological and biochemical examinations

For macroscopic observation of colonies, each strain was streaked out on PDA and CAC (BD Japan, Japan) media and incubated at 25°C for 7 days and 35°C for 4 days, respectively. For microscopic observation, small aliquots of the cells were taken from the slants cultured at 25°C for 5 to 10 days and suspended in a drop of lactophenol cotton blue fluid on a glass slide. Wet specimens were observed under a differential interference contrast microscope (BX51, Olympus, Japan). Carbon assimilation tests were performed with API 20C AUX (bio-Me´rieux, Marcy L’Etoile, France) according to the manufacturer’s instruction.

### PCR analysis

*Prototheca* cells of a colony grown on PDA were harvested with an inoculation loop, and genomic DNA was extracted with a commercial kit (Dr. GenTLE, Takara Bio Inc., Japan) according to the manufacturer’s instruction and dissolved in 50 µl of 2 mM Tris-HCl (pH 8.0) containing 0.4 mM EDTA. PCR was performed by using KOD Plus DNA polymerase (Toyobo, Japan) with 30 cycles of 10 sec at 98°C, 30 sec at 55°C and 3 min at 68°C in the 50μl of reaction mixture containing 1 μl of template DNA, 2mM each of dATP, dCTP, dGTP and dTTP, and 0.3 pmol each of upstream and downstream primers. The primers used are listed in Table 3. PCR products were subjected to electrophoresis in 1.0 or 1.2% agarose gel with Tris-acetate/EDTA buffer and visualized with ethidium bormide staining. As a size marker, the 2-log DNA ladder (New England Biolabs) was used.

**Table 3:** PCR primers used in this study.

No.	Primer name	Sequence	Amplicon	Ref.
①	SSU-F1	5’-AACCTGGTTGATCCTGCCAGTAGTC-3’	SSU rDNA	29
②	SSU-R1	5’- TGATCCTTCTGCAGGTTCACCTACG-3’	SSU rDNA	29
③	ITS5	5’-GGAAGTAAAAGTCGTAACAAGG-3’	ITS and ITS1	30
④	ITS2	5’-GCTGCGTTCTTCATCGATGC-3’	ITS1	30
⑤	ITS3	5’-GCATCGATGAAGAACGCAGC-3’	ITS2	30
⑥	ITS4	5’-TCCTCCGCTTATTGATATGC-3’	ITS and ITS2	30
⑦	28S-F1	5’-AAGCATATCAATAAGCGGAGG-3’	D1/D2 of LSU rDNA	31
⑧	653	5’-GGTCCGTGTTTCAAGACGG-3’	D1/D2 of LSU rDNA	32

### DNA sequencing

Sequencing reactions were performed with a BigDye terminator cycle sequencing kit (Applied Biosystems) according to the manufacturer’s instruction. The reaction products purified with columns (DyeEx 2.0 spin kit, Qiagen) were analysed with PRISM 3130x genetic analyser (Applied Biosystems). For direct sequencing of the PCR products of D1/D2, primers 28SF1 and 635 were used, whereas primers ITS5 and ITS4 were used for the ITS products.

In some cases where direct sequencing of the PCR products generated ambiguous results, the PCR products were cloned in pT7Blue (Novagen) by using the Perfectly Blunt® cloning kit according to the manufacturer’s instruction, and *E. coli* NovaBlue (Novagen) transformed with the plasmid was plated on Luria-Bertani (LB) agar plates containing ampicillin (100 µg/ml), 5-bromo-4-chloro-3-indolyl-β-D-galactoside (40 µg/ml) and isopropylthio-β-D-galactoside (0.5 mM) for the blue-white selection On the next day, white colonies were randomly selected and propagated overnight in 1.5 ml of LB media containing ampicillin (100 µg/ml). Then, plasmid DNA extracted from the grown *E. coli* was used as a template for sequencing. Nucleotide sequences of the cloned DNA were determined by using the conventional M13 (-21) and RV primers. For sequencing clones FL11-0001-1 and -6 derived from the ITS of FL11-0001, additional primers, ITS2, ITS3, Pw-ITS-F2 (5’-GTCCTGTGGCCATGTTCAA-3’), Pw-ITS-R2-1 (5’-GGGCGATTCGCGCATGAAT-3’) and Pw-ITS-R2-2 (5’-GGCAGCAGAGTGCATGCTT-3’), were used. For sequencing clones IFM 56379-1 and -2 derived from the ITS of IFM 56379^T^, primers ITS2, ITS3 and Pw-ITS-R2-3 (5’-CTCCTACGCGGTCAGGTCG-3’) were additionally used. All of the sequences determined in this study are deposited in GenBank, and their assigned accession numbers are shown in Figures 2 and 4 and Table 1.

### Phylogenetic analysis

Nucleotide sequences to be examined were aligned and trimmed by ClustalW and TrimAl (with the “Automated1” option), respectively, at the Phylemon2 web site (http://phylemon2.bioinfo.cipf.es/index.html). The aligned and trimmed sequence data were subjected to the model test for selecting the best substitution model based on Bayesian Information Criterion (BIC) scores. According to the selected model, the phylogenetic trees were built by the Maximum Likelihood method with the bootstrap test (1000 replicates). The model test and the phylogenetic tree generation were performed by using Molecular Evolutionary Genetic Analysis (MEGA) software v. 5.2 [26] downloaded from the MEGA web site (http://www.megasoftware.net/).
